# Social Determinants and Disparities in Active Aging Among Older Taiwanese

**DOI:** 10.3390/ijerph16163005

**Published:** 2019-08-20

**Authors:** Hui-Chuan Hsu, Jersey Liang, Dih-Ling Luh, Chen-Fen Chen, Ying-Wei Wang

**Affiliations:** 1 School of Public Health, Research Center of Health Equity, College of Public Health, Taipei Medical University, 250 Wuxing St., Taipei City 11031, Taiwan; 2 School of Public Health, University of Michigan, 500 S. State Street, Ann Arbor, MI 48109, USA; 3 School of Public Health, Chung Shan Medical University, No.110, Sec.1, Jianguo N. Rd., Taichung City 40201, Taiwan; 4 Department of Social Welfare, Chinese Culture University, No.55, Hwa-Kang Road, Yang-Ming Shan, Taipei City 11114, Taiwan; 5 Ministry of Health and Welfare, No.36, Tacheng St., Taipei City 10341, Taiwan; 6 School of Medicine, Tzu Chi University, Hualien 97004, Taiwan

**Keywords:** active aging, equity, health inequality, older adults, old-age policy

## Abstract

This study assesses equity in active aging across social determinants among older Taiwanese. The data were collected from face-to-face interviews with adults aged 55 years or more in Taiwan in 2017 (*n* = 738). A total of 30 individual-level Taiwan active aging indicators were chosen, and the relationship between social determinants and active aging indicators were analyzed by logistic regression models. Women were more likely to participate in volunteering and other social groups and in lifelong learning activities, whereas men were more likely to be employed, to engage in physical activity, to feel safe from violence, and to use preventive care. Higher education was related to higher employment, social participation, independent living, lifelong learning, and a lower likelihood of poverty and severe cognitive impairment. Those living in rural areas were more likely to be employed, perform physical activity, feel physically safe, have better mental well-being, and have higher social respect and social integration ratings, whereas living in urban areas was related to greater access to medical care, owning assets, less severe cognitive impairment, greater likelihood of using information and communications technology, higher level of education, and higher access to convenient transportation. The significant disparities that exist in active aging may suggest inequality.

## 1. Introduction

Active aging, an issue first raised by the World Health Organization (WHO), is defined as “the process of optimizing opportunities for health, participation and security in order to enhance quality of life as people age” [1]. In the framework for aging and health, the issue of inequalities that underlie this diversity is raised (WHO, 2015). The European Union (EU) and United Nations Economic Commission for Europe (UNECE) subsequently suggested the Active Aging Index (AAI) as an evaluation tool for policy suggestions for European countries. The essential element directly relevant to an active aging policy is “the empowerment of older persons and the promotion of their full participation”, and the four priority goals are to “encourage longer working lives and maintaining work ability; promote participation, non-discrimination and social inclusion of older persons; promote and safeguard dignity, health and independence in older age; and maintain and enhance intergenerational solidarity” [2]. The AAI comprises four domains [2]: employment; participation in society; independent, healthy, and secure living; and active aging capacity and a supportive environment. Both intrinsic and external environments that incorporate all the factors that form the context for old-age life were also emphasized by the WHO [3].

Although the AAI provides measures of active aging, the AAI and its indicators describe only a country’s active aging status and not disparities between different groups in the population. Only the average status is revealed through AAI indicators. In addition, the AAI has also been criticized for not including certain vulnerable groups in active aging policy, such as the homeless, the disabled, and dementia patients. Because the entire population should be targeted for active aging, and not just healthy elders [4], disparities in active aging in society cannot be ignored. This study aims to describe the distribution of active aging indicators in social determinants in the case of Taiwan. When active aging has become a global issue and a trend for aging policy, we expect to indicate disparity issues in active aging for international readers. 

### 1.1. Equity in Active Aging

Whitehead [5] addressed inequity as follows:

The term inequity has a moral and ethical dimension. It refers to differences which are unnecessary and avoidable but, in addition, are also considered unfair and unjust. So, in order to describe a certain situation as inequitable, the cause has to be examined and judged to be unfair in the context of what is going on in the rest of society.

Thus, the causes of the differences in health, well-being, and active aging opportunities are unfair and avoidable. The WHO [6] suggested the following characteristics of frequently assessed stratifications in health inequality monitoring that are usually relevant to policy: place of residence (rural or urban), race or ethnicity, occupation, gender, religion, education, socioeconomic status, and social capital or resources [6].

The socially disadvantaged (e.g., those in lower socioeconomic positions) tend to cluster in groups. Therefore, life opportunities regarding public health are shaped by social determinants, and not just personal behaviors and choices [7]. From a moral and ethical perspective, public health should reduce inequality between social determinant groups. Goldberg [7] cited Powers and Faden’s [8] six dimensions of well-being—that is, health, personal security, reasoning, respect, attachment, and self-determination—whose content should be addressed by justice in public health and health policy. These dimensions also correspond to the content of active aging indicators. From a justice perspective, inequality in active aging should also be addressed and improved through public health and health policy. Although inequality can exist in all domains of active aging, support for vulnerable groups is not comprehensive. For example, Hämäläinen et al. [9] examined the physical activity policies of Denmark, Finland, Romania, and England and, although equity sometimes could be mentioned in the policy issues, no policies specified any additional support for vulnerable groups.

Although the AAI is a composite index describing a country’s active aging status, most of its indicators are measured at the individual level. Therefore, Barslund et al. [10] used data from the Survey of Health, Ageing and Retirement in Europe to compare the inequalities in individual-level active aging indicators by age and gender across 13 European countries. The results indicated that countries with higher AAI scores have greater equality, and countries with lower scores show higher inequality. There were also age differences in the active aging indicators, but the trend was not consistent, and the inequalities in active aging decreased from 2004 to 2013. The differences within a country could be due to variations in economic development, health-care systems, welfare ideologies, aging policies, and other social contextual factors. The following literature review focuses on inequality in active aging indicators in terms of gender, socioeconomic status, and living environment.

Gender inequality issues in health and quality of life have been widely acknowledged. In most patriarchal societies, girls and women usually do not have the same opportunities or social rights as boys and men in education, employment, and elsewhere. Gender roles usually expect girls and women to be the primary family caregivers and to manage the housework. However, as girls and women have been gaining educational opportunities similar to those of boys and men in recent decades, female labor participation has been increasing in many cultures, and the care-giving role therefore also needs to be changed [11]. Past research also provides much evidence of gender inequality. Gender inequality among older adults has been found in cases of multimorbidity [12], healthy life expectancy and physical function [13], income and assets [14], physical activity interventions [15], community-based health insurance enrollment [16], Internet use [17], mental health [18], and employment and pensions [19]. However, the results regarding differences in the likelihood of social participation between male and female elderly persons have been mixed [20,21], with females potentially receiving greater social support than males, even in resource-deprived areas [22]. The promotion of active aging to prevent gender inequality in life chances can start as early as in the womb, as a fetus, to the end of life, over the entire course of a person’s life [23]. At the same time, gender roles and expectations have also changed, because gender equality has improved in recent decades. Gender effects on active aging are expected to be more equal. 

Social stratification affects life opportunities and thus reveals the effects of inequality on health. Occupation, education, and income are usually used to measure socioeconomic status, and employment, income, and education are all AAI indicators. Socioeconomic status is related to self-rated health, healthy behavior, life satisfaction, and quality of life among older Europeans [24]. Lower education is related to a higher likelihood of multimorbidity, but the income effect is mixed [25]. For lower-income countries without universal health insurance, education and income are related to enrollment in community-based health insurance programs, thus affecting access to health care [16,26]. The utilization of doctor visits is positively related to higher income across 18 Organisation for Economic Co-operation and Development (OECD) countries with the same health needs, indicating income inequality in health-care utilization [27]. Socioeconomic status or the availability of health insurance among older adults is related to health-care utilization, unmet needs, and delayed health care [28,29,30].

Education is the most common socioeconomic indicator in health research, because it represents both material and non-material resources [31]. Education is related to both healthy and risky behaviors, such as smoking [32], whereas financial strain is related to greater incidence of mental-health disorders [33]. Socioeconomic status is also related to many other active aging indicators among older adults, such as use of the Internet [34,35]. A Brazilian study has found educational inequalities in health and active aging indicators, such as self-rated health, health-care utilization, neighborhood of residence, and physical safety [36]. Older adults who have either higher education or higher socioeconomic status are more likely to engage in social participation [37], whereas an individual’s lower socioeconomic status is related to lower perceived neighborhood safety [38], and lower neighborhood socioeconomic status is related to violent injury [39].

Living areas also affect active aging, through the availability and accessibility of resources. For example, the incidence of health examinations and health-care utilization is higher in affluent areas than in deprived areas [30,40,41]. Walkability for the elderly and their demands and supplier-loading factors determine geographical accessibility to community-based care [42]. Internet infrastructure affects Internet-use behavior [34], and rural areas usually have fewer such resources than urban areas. However, contextual factors do not always favor urbanization. Those living around more green spaces have lower risk of cardiovascular disease [43]. The findings about the association between living areas and mental health are mixed. Some studies reported that the elderly living in more disadvantaged or rural areas are subject to more depressive symptoms [44,45], whereas other research found an association between anxiety- and stress-related disorders and urbanization [46]. 

Although resource inequality affects health, perceived deprivation also plays a role. Neighborhoods that reflect income inequality and income deprivation are related to a higher incidence of mental disorders [47]. This finding supports relative deprivation theory [48]: as inequality in a society increases, the feeling of unfairness affects mental well-being to a greater extent. Thus, mental wellbeing and social integration are expected to be lower in more-deprived neighborhoods (usually in urban areas). 

### 1.2. Background

Taiwan, in the Republic of China, has a population of 23.57 million. The gross domestic product per capita was $25,893 in 2016. Taiwan is a fast-aging society. The older population (aged 65 or older) comprised 7% of the total population in 1993, and it doubled to 14.1% in 2017 [49]. Taiwan’s National Health Insurance system was launched in 1995, which significantly improved access to health care. Taiwan’s Gender Inequality Index was 73 in 2012, indicating a rather equitable society compared with OECD countries. Due to the country’s history of colonization by Japan and civil war in Mainland China, older cohorts did not have good educational opportunities; however, Taiwan’s level of education has improved in recent years.

According to the report, “Health Inequalities in Taiwan”, by the Health Promotion Administration, Ministry of Health and Welfare, there are also inequities in the social determinants of health in Taiwan: Gaps in life expectancy between the most and least deprived quintiles were 6.4 years for men and 3.5 years for women in Taiwanese townships. The mortality rate among blue-collar workers was 84% higher than among white-collar workers. An education equal to or below secondary education was related to a shorter life expectancy. Education was also related to higher rates of morbidity and disability. Household income was related to life satisfaction. Therefore, disparities in these social determinants are expected not just in health but also in all active aging indicators.

This study focuses on disparities in residence, gender, and education in active aging in Taiwan. We hypothesize that males, higher education (as indicator of socioeconomic status), being married (having more social capital), and urbanization are associated with a higher possibility of active aging.

## 2. Materials and Methods

### 2.1. Data and the Sample

A face-to-face survey was conducted in 2017 to collect data on active aging indicators in Taiwan [50]. Four cities were selected: New Taipei City, in the northern region; Taichung, in the central region; Kaohsiung, in the southern region; and Yilan, in the eastern region. In each city, one urban district and one rural district were selected for active aging indicator testing. Eight districts were selected in total and, in each district, 250 persons were selected for the survey. The sampling method was based on the household registration system and included those aged 55 or older living in the eight districts selected, and probability proportional to size sampling was applied to each district at the township, neighborhood (i.e., “Lin” in Chinese), and individual levels. Of the 2,131 persons in the sample, 738 completed the survey, for a completion rate of 34.6%. The interviewers were recruited from the survey region and had experiences of being interviewers. All the interviewers were trained by the principal investigator of this project before the survey, and research ethical education, study background, and interview standardization were provided during the training. 

All the participants were informed of the study’s purpose and content, their rights and free participation in the study, and the confidentiality and data protection policy of the study before they agreed to participate. Informed consent documents were signed before the interviews. The study was approved by the Research Ethics Committee of the China Medical University and Hospital (CRREC-105-025) and Asia University (No.10503006).

### 2.2. Measures

Taiwan’s Active Aging Index (TAAI) was constructed for policy monitoring and evaluation, as described elsewhere [50]. The TAAI indicators address the same four domains as the AAI. This study, however, uses only individual-level indicators to analyze the disparity in active aging. All the variables are defined to show the percentage of the indicators. The definitions of the variables are as follows.

#### 2.2.1. Employment

The participants were asked if they were working (at least one hour per week). Taiwan’s Active Aging Index uses employment rates stratified by age as indicators, with the age ranges 55–59, 60–64, 65–69, 70–74, and 75 and above.

#### 2.2.2. Social Participation

Five indicators were used to measure social participation: (1) volunteering, that is, participating in any form of volunteering in the last 12 months; (2) caring for children or grandchildren at least once per week; (3) caring for elderly or disabled family members at least once per week; (4) political participation in the past 12 months; and (5) participation in other social groups at least once per month in social groups other than those previously mentioned.

#### 2.2.3. Independent, Healthy, and Secure Living

A total of 12 yes/no variables were used in this domain:

1. Physical activity was defined as performing exercise or physical activity at least five times per week.
2. Access to health and dental care was defined as having no unmet medical or dental-care needs in the past 12 months.
3. Independent living arrangements (for those aged 75+) were defined as the participant living alone or living only with a partner/spouse.
4. Absence of poverty risk (for those aged 65+): In this study, we used the household income and number of household members to calculate the equivalized personal risk of poverty, and the risk of poverty was defined as an equivalized individual income below the lowest living standard in 2017.
5. The absence of severe material deprivation was defined as having at least six of the 12 items commonly owned by Taiwanese households.
6. Personal safety (from violence or crime) was defined as the absence of fear of violence or crime in the household or neighborhood.
7. Personal safety from accidents or injury was defined as the absence of fear of being injured by traffic, falls, or other accidents in the household and in the neighborhood.
8. Lifelong learning (for those aged 55–74) was defined as participation in any kind of education or training in the past month.
9. Physical function independence was measured by no or only minor difficulty performing activities of daily life (ADLs).
10. The absence of severe cognitive impairment: Mini-Mental State Examination [51] in this study, and absence of severe cognitive impairment was indicated by scores of 22 or above, according to the recommendations for the Chinese population [52].
11. The absence of depressive symptoms was measured by the Center for Epidemiologic Studies Depression Scale (CES-D) 10-item scale [53], where scores of seven or less were defined as the absence of depression [54].
12. Primary prevention utilization (for those aged 65+) was defined as the use of flu vaccination and health checkups in the past year. These two primary prevention health services are free in Taiwan for those aged 65 and older.

The total score for the 12 variables, ranging from zero to 12, was also used in this domain.

#### 2.2.4. Capacity of Active Aging and Supportive Environment

1. Mental well-being was measured by the WHO-5 Well-Being Index [55], with a raw score of 0 to 25. Scores of 14 and above were defined as indicating positive mental well-being.
2. Use of information and communications technology (ICT) was defined as the use of any kind of device with Internet access at least once per week.
3. Social connectedness was defined as informal interactions with family or friends (not including business) at least once per week.
4. Transportation accessibility was defined as the ability to access typical locations in one’s life circle by any kind of transportation.
5. Transportation convenience was measured by rating multiple items, including pavement smoothness, crossroad convenience, traffic light/sign clarity, and convenience in taking the bus (frequency, timeliness, safety, good driver attitude). Ratings of good or excellent were defined as indicating convenient transportation.
6. Barrier-free spaces were defined to indicate the absence of barriers in building and public spaces affecting social participation outdoors or at home in going up and down stairs in the past 12 months.
7. Social integration and social respect were measured by community activities that consider accessibility for older adults, social activities and commodities that meet older adults’ needs, and a positive public view of older people. When all three items are positive, social integration and respect are present.
8. Educational attainment (i.e., high school or higher education) was one of the TAAI indicators. In this study, educational level was also defined as a social determinant. Therefore, the total score of the seven indicators (each one scoring 0 or 1, indicating no or yes, respectively) was also used to define the score in this domain, which ranged from 0 to 7.

#### 2.2.5. Demographics and Controlling Variables 

Social determinants included age, gender, educational levels (illiterate or non-formal education, elementary school, primary high school, senior high school, college or university, and above), area of residence (rural/urban), and regions (northern, central, southern, and eastern). In addition, marital status (having spouse or not) was also included in the model to represent social capital.

### 2.3. Analysis

Descriptive analysis and chi-squared tests were used to determine the distributions of the active aging indicators. Disparities in the social determinants for each indicator were examined by logistic regression analysis.

## 3. Results

Table 1 shows the sample description and distribution of active aging indicators by demographic variables. Among the 27 individual-level Taiwan active aging indicators, age differences were found in employment, childcare, political participation, physical disability, cognitive impairment, depressive symptoms, preventive care utilization, mental well-being, ICT use, educational achievement, transportation accessibility, and barrier-free spaces. Most age differences in the indicators favored younger participants, except in political participation and preventive-care utilization. Gender differences were found in employment, physical activity, owning assets, no depressive symptoms, educational achievement, and barrier-free spaces, for which men reported better than women. Educational disparities also affected employment, volunteering, childcare, participation in other social groups, independent living, poverty risk, assets, personal safety (from violence), lifelong learning, cognitive impairment, depressive symptoms, ICT use, social connectedness, transportation accessibility, and barrier-free spaces. Generally, the higher one’s education, the higher the likelihood of active aging, except regarding childcare and personal safety from violence. Rural–urban disparities arose in many active aging indicators. Participants living in urban areas of residence scored higher in volunteering, childcare, participation in other social groups, medical accessibility, owning assets, safety from violence, lifelong learning, no severe cognitive impairment, ICT use, educational achievement, transportation accessibility, transportation convenience, and barrier-free spaces, whereas participants living in rural areas showed higher percentages in employment, physical activity, mental well-being, and social integration and respect. There were also differences in active aging indicators across regions and marital status.

Table 2, Table 3, Table 4 and Table 5 show the factors related to the indicators and domains by controlling for other variables through logistic or linear regression. Table 2 shows the results of the Employment domain by age groups and total employment. Personal factors did not significantly affect the employment rate for participants aged 55–59, 70–74, and 75 and older. Among the participants aged 60–64, having higher education (OR = 1.313), residence in a rural area (OR = 2.703), and residence in the central region of Taiwan (OR = 2.592), were more likely to be associated with employment. Among individuals aged 65–69, men (OR = 3.678) were more likely to be employed, but those living in the southern region (OR = 0.260) were less likely to work. Social determinants were not significant for the age group 55–59 or the age groups 70 and above.

Table 3 shows the disparities in the Social Participation domain. Participants who were older than 75 years (OR = 1.839), female (OR = 0.565), and had a higher level of education (OR = 1.283) were more likely to participate in volunteering. Those who were younger (OR = 0.867 for age 65–74, and OR = 0.530 for age 75 or more) and had a spouse (OR = 1.829) were more likely to engage in childcare. Having a higher education (OR = 1.350) and living in the southern region of Taiwan (OR = 2.445) were associated with a higher possibility of caring for elderly or disabled family members. Individuals who were older than 75 years (OR = 7.958), had a higher education (OR = 2.194), and lived in a rural area (OR = 5.327) were more likely to be engaged in politics. Women (OR = 0.644) and higher-educated (OR = 1.282) participants were more likely to participate in other social groups.

Table 4 shows the factors related to Independence, Health, and a Secure Living domain and indicators. Being older was related to a higher likelihood of physical activity, independent living, absence of poverty risk, and primary prevention care utilization, whereas being younger was more strongly associated with the absence of severe material deprivation, physical safety from injury or accidents, absence of severe physical disability, and absence of severe cognitive impairment. Although men exhibited a higher likelihood of physical activity (OR = 1.516) and physical safety from violence (OR = 3.462), women had a higher likelihood of engaging in lifelong learning (OR of males = 0.546). Having higher education was associated with independent living, absence of poverty risk, lifelong learning, absence of cognitive impairment, and absence of depressive symptoms, but was also related to a lower rating in physical safety from violence. Participants living in rural areas were more likely to perform physical activity (OR = 2.050) but less likely to have access to medical care (OR = 0.074), to own assets (OR = 0.408), to feel safe from violence (OR = 0.253), and to be free of severe cognitive impairment (OR = 0.214). There were regional differences in independent living, absence of poverty risk, owning assets, safety from violence and injury, and the use of primary preventive care.

Table 5 shows the factors of the Capacity of Active Aging and Supportive Environments domain. Generally, being older was related to lower chances of positive mental well-being, lower use of ICT, lower educational attainment, lower transportation accessibility, and lower ratings regarding barrier-free spaces. Higher education was related to higher chances of using ICT, greater social connectedness, better transportation accessibility, and greater enjoyment of barrier-free spaces, but was also rated to lower transportation convenience. Participants living in rural areas were more likely to have positive mental well-being (OR = 10.234) and higher social integration and respect (OR = 7.397), but also had lower levels of ICT use (OR = 0.277), transportation convenience (OR = 0.270), and barrier-free space (OR = 0.506). There were also regional differences: Compared to the northern region, participants living in the southern and eastern regions showed lower capacities and a lower supportive environment. 

## 4. Discussion

This study examined the distribution of active aging indicators among older adults in Taiwan. Disparities in active aging were found in age, gender, and education, between rural and urban areas, and across regions. The disparities of age, gender, education, and living area did not show consistent trends in all the active aging indicators.

### 4.1. Gender Disparity

Generally, the gender differences in active aging indicators in Taiwan were not as large as in most OECD countries [2]. Men had better opportunities than women in employment, education, physical activity, and physical safety from violence. The advantages of men over women were likely related to work and education opportunities among older adults. The finding that older men engaged in more physical activity than older women is consistent with previous research [56,57]. Although men still performed better in terms of health-related variables (physical function, cognitive impairment, and mental well-being), the gender differences were not significant when we controlled for the other variables. One reason for this is that the respondents could be healthier than the others, and the more severely disabled (more likely to be women) did not participate in the survey. The other explanation is that gender differences in health have decreased in recent years due to the National Health Insurance system, and education levels have increased among both genders. Women are not always disadvantaged.

In this study, women showed greater participation in volunteering, other social groups, and lifelong learning. There were no significant differences in terms of taking care of children or the elderly. Previous studies indicated gender differences in different kinds of social group participation [58]. Men were more likely to participate in social groups related to occupations and employment, whereas women were more likely to participate in cultural and learning activities. After the Community Care Center Plan was implemented in Taiwan in 2005, learning, social, and recreational activities have been widely provided among older adults [59]. The policy provides good opportunities for women who did not have a chance to become well educated to participate in lifelong learning and volunteering activities. Gender disparity in social group participation can thus be reduced. The lack of gender differences among family caregivers was unexpected, since women are usually more likely to be family caregivers than men are [60]. Possible explanations are that the co-residence rates of the elderly with their adult children have greatly decreased, as has Taiwan’s birth rate, and older adults thus have fewer chances to care for children than before. Additionally, the primary family caregivers of disabled older adults in Taiwan are more likely to be adult children or daughters-in-law, who are usually middle-aged adults. Gender differences in active aging are expected to be further reduced in future decades, as gender equality in education, employment, and social participation in Taiwan improve.

### 4.2. Educational Disparity

Higher-educated older adults scored better in most kinds of employment at age 60–64, in social participation (volunteering, caring for older or disabled family members, political participation, participation in other social groups), independent living, absence of poverty risk, lifelong learning, absence of severe cognitive impairment, ICT use, social connectedness, and transportation accessibility. Higher education provides more advantages and social capital in cases of active aging, consistent with previous findings [36,37]. However, higher-educated older adults scored lower in physical safety from violence and in transportation convenience, which is inconsistent with previous research [38,39]. It is possible that individuals with higher education have higher standards and expectations of personal safety and more convenience in terms of transportation system, or other confounding factors related to education and perceived safety.

### 4.3. Disparity in Residential Areas

The most significant disparities in active aging indicators involved area of residence. Urbanization is associated with greater access to medical care, a higher likelihood of having assets, feeling safe from violence, the absence of cognitive impairment, and ICT use. The Internet infrastructure and medical resources are better in urban areas, as noted in previous research [30,34,40,41,42]. Additionally, individuals of higher socioeconomic status are more likely to live in urban areas, thus associating urbanization with better economic status or cognitive function. However, those living in rural areas had higher employment rates and greater political participation, were more physically active, and had better mental well-being and higher social integration and social respect. One explanation is that adults living in rural areas are more likely to work in a primary industry (e.g., farming), where there is no definite retirement age and older adults can continue working as long as they want. Rural areas are also associated with a more active lifestyle. The other explanation is that the social atmosphere in rural areas is friendlier and that urbanization is associated with loneliness, especially for older adults [61]. 

Previous research indicated that urbanization is usually associated with various mental conditions [46], and income inequality can cause feelings of deprivation in urban areas [47]. The WHO [62] defines an age-friendly city according to eight domains: housing, transportation, outdoor spaces and buildings, community support and health services, communication and information, civic participation and employment, respect and social inclusion, and social participation. An age-friendly city consists of not only the “hardware” (infrastructure and services) but also the “software” (psychological and social capital). Disparities in hardware between areas can be compensated for by resources. However, disparities in software are not easily corrected in a short time; community involvement and participation are necessary.

### 4.4. Age Differences

Age differences (especially among those aged 75 or more) were found for employment, some kinds of social participation (childcare), the absence of economic risks, feeling physically safe from violence, the absence of severe cognitive impairment, mental well-being, the use of ICT, better access to transportation, and fewer limitations in barrier-free spaces. Older individuals are expected to exhibit a poorer performance in all active aging indicators, due to frailty and lack of ability to participate, consistent with previous research [10,63]. Age discrimination can also prevent older adults from participating in social affairs and affect their perceptions of well-being [64]. However, the Taiwanese data showed that being older was related to greater participation in volunteering, politics, physical activity, independent living, and preventive care. It is possible that the older elderly have fewer responsibilities and can thus allocate more time to social participation. Also, traditionally, Chinese culture respects the elderly and their social participation is promoted in current Taiwanese society. The elderly could also be aware of the importance of maintaining physical function and are thus willing to engage more in physical activity. The use of preventive care in both flu vaccinations and health checkups, which are supported by the government, is free for adults aged 65 years and older. 

### 4.5. Limitations

This study does have some limitations, however. First, the data were collected based on selected cities and areas and therefore cannot be generalized to the entire older population of Taiwan. Second, the data were cross-sectional. Causal relations among social determinants in active aging disparities could not be confirmed. Third, confounders associated with disparities in gender, education, and living areas that could not be separated or not included in the data (such as occupation and morbidity).

## 5. Conclusions

There are active aging disparities in gender, education, and living areas for older adults in Taiwan. Some disparities, such as education and employment, are expected to be decreased in future cohorts. We suggest that aging policies should be more gender sensitive to eliminate gender disparities. Disparities due to socioeconomic status should be reduced by offering more equal opportunities in all domains of aging life, and the cumulative disadvantages due to socioeconomic inequality should be eliminated by social policy starting in early life. Area disparities can be reduced through the input of infrastructure in rural areas and community involvement in urban areas. We suggest long-term monitoring of active aging to evaluate aging policies. Regarding the critics of neglecting vulnerable populations in active aging, the intervention in vulnerable populations (the homeless, the disabled, and people living with dementia) may apply the indicators in TAAI (i.e., owning assets, no severe physical disability, and no severe cognitive impairment) for policy evaluation. Through regular data of active aging indicators, the government can also monitor the opportunities of active aging for these vulnerable populations, and provide specific or tailored services for them. In addition, multi-morbidity may affect active aging performance [54,65,66,67]. A universal policy or program of health promotion and prevention of multi-morbidity would be a fundamental solution to reduce disparity in the population, such as the health policy of flu vaccination and health checkup for older adults in Taiwan. We also suggest that longitudinal studies on the effects of disparities of social determinants on active aging should be conducted in the future. 

## Figures and Tables

**Table 1:** Sample description and distribution of active aging indicators by social determinants (%).

Social Determinants	Active Aging Indicators														
	Sample % (*n* = 738)	Employment	Volunteering	Caring for Children	Caring for Elderly/Disabled	Politic Participation	Other Social Group Participation	Physical Activity	Medical Access	Independent Living	Absence of Poverty Risk	Absence of Material Deprivation		Owning Assets	
Total	100.0 (738)	34.8	15.9	19.5	5.3	3.1	11.5	70.6	98.0	30.9	83.1	98.8		82.9	
		***		***		*									
Age 55–59	15.2 (112)	69.6	13.4	23.2	6.3	0.0	8.0	59.8	96.4	21.4	83.8	100.0		86.6	
Age 60–64	23.6 (174)	48.3	17.8	28.2	6.9	5.2	15.5	69.0	95.4	28.7	91.0	99.4		83.9	
Age 65–69	18.7 (138)	29.0	15.9	23.9	4.3	0.7	13.8	73.9	98.6	35.5	80.0	100.0		84.8	
Age 70–74	16.4 (121)	25.6	13.2	15.7	1.7	3.3	10.7	75.2	100.0	36.4	75.0	97.5		81.8	
Age 75+	26.1 (193)	12.4	17.1	8.8	6.2	4.7	8.8	73.1	99.5	31.6	83.1	97.4		79.3	
Gender		***						*						***	
Male	48.8 (360)	42.2	13.8	18.3	5.6	3.6	10.6	74.4	98.1	33.3	82.0	98.3		90.3	
Female	51.2 (378)	27.8	17.7	20.6	5.0	2.6	12.4	66.9	97.9	28.6	84.4	99.2		75.9	
Education		***	*	***			***			***	*			***	
Illiterate/non-formal	22.0 (162)	20.4	9.3	8.6	3.7	1.9	3.7	72.2	97.5	29.0	82.0	96.9		66.0	
Elementary school	40.4 (298)	32.9	14.4	21.1	5.0	3.0	9.1	70.1	97.3	24.8	78.2	99.7		87.6	
Primary high school	15.4 (114)	52.6	19.3	32.5	3.5	2.6	18.4	69.3	98.2	28.9	86.3	100.0		83.3	
Senior high school	13.7 (101)	39.6	24.8	17.8	6.9	5.0	19.8	71.3	100.0	37.6	87.5	98.0		91.1	
College/University+	8.5 (63)	41.3	19.0	19.0	11.1	4.8	17.5	69.8	98.4	57.1	95.5	98.4		90.5	
Residence area		**	*	*			***	**	**					***	
Urban	50.9 (376)	29.0	18.6	22.9	6.1	2.1	16.5	64.9	99.7	32.4	81.9	98.9		88.8	
Rural	49.1 (362)	40.9	13.0	16.0	4.4	4.1	6.4	76.5	96.1	29.3	84.5	98.6		76.8	
Region		*	***			***	**				**			***	
Northern	24.9 (184)	36.4	12.5	20.1	3.8	1.6	8.7	69.6	98.9	26.6	88.1	100.0		76.1	
Central	23.8 (176)	42.6	18.8	22.7	2.8	9.7	8.0	63.6	97.2	27.8	91.3	98.9		97.7	
Southern	24.8 (183)	30.6	25.1	20.2	8.7	1.1	19.1	74.3	97.8	33.9	82.5	97.3		90.2	
Eastern	26.4 (195)	30.3	7.7	15.4	5.6	0.5	10.3	74.4	97.9	34.9	74.2	99.0		69.2	
Marital status		***		*					**			*		*	
Never married	1.8 (13)	38.5	15.4	7.7	0.0	7.7	7.7	69.2	84.6	46.2	100.0	92.3		53.8	
Married	72.4 (534)	39.7	15.9	22.1	6.4	3.4	11.4	71.9	98.1	32.4	82.6	99.3		84.3	
Divorce/Widow/Other	25.8 (191)	20.9	15.7	13.1	2.6	2.1	12.0	69.6	98.4	25.7	83.1	97.9		81.2	
**Social Determinants**	**Active Aging Indicators**														
	**Safety (from Violence)**	**Safety (from Injury or Accidents)**	**Lifelong Learning**	**Absence of Severe Physical Disability**	**Absence of Severe Cognitive Impairment**	**Absence of Depressive Symptoms**	**Primary Prevention Utilization**	**Mental Well-being**	**ICT use**	**Social Connectedness**	**Educational Achievement**	**Transportation Accessibility**	**Transportation Convenience**	**Barrier-free Space**	**Social Integration and Respect**
Total	89.7	75.2	11.2	97.7	75.2	81.4	35.4	79.1	42.8	81.7	22.2	91.7	76.1	84.4	75.2
				**	***	***	**	**	***		***	***		***	
Age 55–59	92.9	76.8	15.2	99.1	95.5	86.6	25.0	83.0	73.2	84.8	38.4	97.3	82.9	94.6	70.5
Age 60–64	89.1	79.3	13.2	100.0	84.5	86.2	29.9	87.4	63.2	84.5	29.9	98.9	83.1	90.8	77.6
Age 65–69	90.6	74.6	10.1	97.8	79.9	88.4	37.0	81.9	40.6	87.0	21.7	92.8	65.2	84.8	76.8
Age 70–74	90.9	77.7	11.6	98.3	74.4	79.3	33.1	70.2	30.6	76.9	15.7	94.2	80.0	86.8	71.9
Age 75+	87.0	69.4	7.8	94.3	52.8	70.5	46.6	73.1	16.1	76.7	10.4	79.8	70.8	71.0	76.7
Gender						*					***			*	
Male	91.4	74.7	10.6	98.1	78.1	85.0	37.5	80.3	45.0	82.5	28.9	92.8	72.9	87.2	76.7
Female	88.1	75.7	11.9	97.4	72.5	78.0	33.3	78.0	40.7	81.0	15.9	90.7	78.1	81.7	73.8
Education	*		***		***	***			***	*		***		***	
Illiterate/non-formal	93.2	77.8	3.1	95.1	34.6	69.8	40.1	81.5	7.4	75.9	---	79.6	72.7	64.8	80.2
Elementary school	85.2	73.2	5.0	97.7	78.5	82.6	34.6	77.5	31.2	79.5	---	92.6	76.5	85.9	77.2
Primary high school	95.6	80.7	17.5	100.9	93.0	85.1	36.8	83.3	69.3	87.7	---	96.5	75.7	89.5	71.1
Senior high school	90.1	67.3	24.8	98.0	95.0	87.1	28.7	77.2	74.3	86.1	---	98.0	82.9	98.0	68.3
College/University+	90.5	81.9	28.6	100.0	100.0	90.5	34.9	76.2	90.5	88.9	---	100.0	72.7	96.8	71.4
Towns	***		***		***			***	***		***	**	*	***	***
Urban	93.6	77.5	17.3	98.1	90.7	84.0	33.5	68.1	62.5	83.0	36.7	94.7	82.6	91.8	61.4
Rural	85.6	72.8	5.0	97.2	59.1	78.7	37.3	90.6	22.4	80.4	7.2	88.7	68.9	76.8	89.5
Region	***	***	**		**	*		***	***	***	*		***	***	**
Northern	94.0	84.2	9.2	96.7	70.1	84.8	38.6	87.0	48.4	82.1	17.4	90.2	85.0	80.4	81.5
Central	67.6	44.9	17.6	98.3	85.8	75.6	34.7	86.9	56.3	65.9	27.8	90.9	79.1	90.3	78.4
Southern	96.7	72.7	13.1	98.4	72.1	85.6	35.5	89.6	36.1	85.8	25.7	93.4	52.3	90.7	64.5
Eastern	99.0	96.4	5.6	97.4	73.3	79.5	32.8	54.9	31.8	91.8	18.5	92.3	70.4	76.9	76.4
Marital status				*	***	**		**	***		**	***			
Never married	76.9	53.8	7.7	92.3	76.9	76.9	30.8	69.2	46.2	84.6	38.5	76.9	66.7	84.6	69.2
Married	90.4	75.7	12.2	98.5	79.0	84.8	35.2	82.4	47.4	82.8	24.7	94.4	77.7	86.1	76.4
Divorce/Widow/Other	88.5	75.4	8.9	95.8	64.4	72.3	36.1	70.7	29.8	78.5	14.1	85.3	72.5	79.6	72.3

Note: Data were unweighted. Chi-square test, * *p* < 0.05, ** *p* < 0.01, *** *p* < 0.001.

**Table 2:** Employment indicators and social determinants by logistic regression.

Social Determinants	Work (Age 55–59) (*n* = 112)	Work (Age 60–64) (*n* = 174)	Work (Age 65–69) (*n* = 138)	Work (Age 70–74) (*n* = 121)	Work (Age 75+) (*n* = 193)
Sex (male)	1.951	1.660	3.678 *	2.241	2.559
Education	1.360	1.313 *	0.970	0.70	0.878
Marital status (having spouse)	0.570	1.198	1.661	1.358	6.241
Residence (rural)	0.687	2.703 *	1.840	2.772	2.929
Region (central)	0.902	2.592 *	0.657	1.846	0.745
Region (southern)	0.429	0.738	0.260 *	1.064	0.587
Region (eastern)	0.909	1.757	0.381	1.310	0.407
Model summary	–2LL = 186.778, χ^2^ = 16.318 (df = 7) *	–2LL = 269.303, χ^2^ = 24.789 (df = 7) ***	–2LL = 116.632, χ^2^ = 15.491 (df = 7) *	–2LL = 84.060, χ^2^ = 7.820 (df = 7)	–2LL = 60.078, χ^2^ = 12.064 (df = 7)

Note: Data were weighted. Reference groups: sex (female), marital status (no spouse), region (northern), residence (urban). Constants are omitted. Ordinal variables included: education and household income. Logistic regression analysis was conducted. –2LL = –2 log likelihood. * *p* < 0.05, ** *p* < 0.01, *** *p* < 0.001.

**Table 3:** Social participation indicators and social determinants by logistic regression.

Social Determinants	Volunteering	Caring for Children	Caring for Older or Disabled Family	Political Participation	Other Social Group Participation
Age 65–74	1.190	0.867	0.575	0.670	1.262
Age 75+	1.839 *	0.530 *	1.371	7.958 **	0.829
Sex (male)	0.565 **	0.987	0.550	0.663	0.644 *
Education	1.283 **	0.898	1.350 *	2.194 **	1.282 **
Marital status (having spouse)	1.233	1.829 *	2.168	3.020	1.083
Residence (rural)	0.901	0.669	0.961	5.327 *	0.481
Region (central)	1.055	1.109	0.687	1.674	0.743
Region (southern)	1.065	0.893	2.445 *	0.912	1.220
Region (eastern)	0.578	0.496 *	2.219	0.368	1.278
Model summary	–2LL = 668.5990, χ^2^ = 22.326 (df = 9) **	–2LL = 760.371, χ^2^ = 29.338 (df = 9) **	–2LL = 314.476, χ^2^ = 22.530 (df = 9) **	–2LL = 138.130, χ^2^ 27.679 (df = 9) **	–2LL = 605.006, χ^2^ = 24.557 (df = 9) **

Note: *N* = 738. Data were weighted. Reference groups: age group (age 55–64), sex (female), marital status (no spouse), region (northern), residence (urban). Constants are omitted. Ordinal variables included: education and household income. Logistic regression analysis was conducted. –2LL = –2 log likelihood. * *p* < 0.05, ** *p* < 0.01, *** *p* < 0.001.

**Table 4:** Independent, healthy, and secure living indicators and social determinants by logistic regression.

Social Determinants	Physical Activity	Medical Care Accessibility	Independent Living	Absence of Poverty Risk	No Severe Material Deprivation	Owning Assets	Physical Safety (from Violence)	Physical Safety (from Injury or Accidents)	Lifelong Learning	Absence of Severe Physical Disability	Absence of Severe Cognitive Impairment	Absence of Depressive Symptoms	Using Preventive Care
Age 65–74	1.761 **	11.405	2.170 ***	0.546 *	2.064	1.037	0.977	0.847	1.077	0.092	0.545	0.883	1.226
Age 75+	2.210 **	22.645	1.994 **	1.339	0.081 **	0.626	0.725	0.396 **	1.074	0.031 *	0.227 ***	0.503 *	2.082 **
Sex (male)	1.516 *	1.968	0.830	0.769	0.343	1.348	3.462 ***	1.034	0.546 **	0.843	0.696	1.094	1.175
Education	1.103	1.231	1.556 ***	1.683 ***	0.613	1.113	0.717 **	0.977	1.695 ***	1.460	2.115 ***	1.229 *	1.086
Marital status (having spouse)	1.183	1.110	1.512 ***	0.599	1.769	2.168 **	0.998	0.755	1.557	1.964	2.223 *	1.854 **	1.003
Residence (rural)	2.050 **	0.074 **	1.397	1.604	0.848	0.408 **	0.253 **	0.805	0.455	1.217	0.214 ***	0.903	1.127
Region (central)	0.853	0.372	1.507	0.800	0.000	6.673 ***	0.420 *	0.462 **	1.497	7.884	2.171	0.662	1.682 *
Region (southern)	1.195	0.234	1.730	0.440	0.000	1.248	1.304	0.490 **	1.055	2.146	0.545	0.888	0.848
Region (eastern)	1.094	0.493	2.590 ***	0.134 ***	0.000	1.816	39.604 *	12.887 ***	0.640	1.852	1.921	1.140	0.658
Model summary	–2LL = 915.185, χ^2^ = 34.484 (df = 9) ***	–2LL = 55.028, χ^2^ = 20.007 (df = 10) *	–2LL = 840.524, χ^2^ = 77.950 (df = 9) ***	–2LL = 383.842, χ^2^ = 68.295 (df = 9) ***	–2LL = 59.500, χ^2^ = 25.115 (df = 9) **	–2LL = 488.667, χ^2^ = 36.318 (df = 9) ***	–2LL = 323.057, χ^2^ = 65.171 (df = 9) ***	–2LL = 683.539, χ^2^ = 109.090 (df = 9) ***	–2LL = 582.791, χ^2^ = 67.830 (df = 9) ***	–2LL = 99.089, χ^2^ = 28.190 (df = 9) **	–2LL = 353.962, χ^2^ = 184.491 (df = 9) ***	–2LL = 605.936, χ^2^ = 41.153 (df = 9) ***	–2LL = 910.451, χ^2^ = 29.818 (df = 9) ***

Note: *N* = 738. Data were weighted. Reference groups: age group (age 55–64), sex (female), marital status (no spouse), region (northern), residence (urban). Constants are omitted. Ordinal variables included: education and household income. Logistic regression analysis was conducted. –2LL = –2 log likelihood. * *p* < 0.05, ** *p* < 0.01, *** *p* < 0.001.

**Table 5:** Capacity of active aging and supportive environment indicators and social determinants by logistic regression.

Social Determinants	Mental Well-Being	Use of ICT	Social Connectedness	Educational Attainment	Transportation Accessibility	Transportation Convenience	Barrier-Free Space	Social Integration and Respect
Age 65–74	0.553 *	0.340 ***	0.967	0.556 *	0.508	0.656	0.601	0.975
Age 75+	0.373 **	0.205 ***	0.523 *	0.354 ***	0.125 ***	1.037	0.293 **	0.781
Sex (male)	0.873	1.000	0.913	2.354 ***	0.487	0.661	0.858	0.917
Education	1.116	2.030 ***	1.261 **	---	1.604 **	0.742 *	1.614 ***	1.148
Marital status (having spouse)	1.539	1.221	0.980	1.579 *	2.684 *	1.222	1.261	1.070
Residence (rural)	10.234 ***	0.277 ***	1.143	0.145 ***	0.904	0.270 *	0.506 *	7.397 ***
Region (central)	1.284	1.293	0.678	2.092 **	1.200	1.330	0.807	1.152
Region (southern)	1.033	0.370 ***	1.447	2.087 **	1.761	0.334 **	0.808	0.213 ***
Region (eastern)	0.065 ***	0.279 ***	3.301 ***	1.383	2.827 *	0.993	1.784	0.925
Model summary	–2LL = 610.400, χ^2^ = 261.792 (df = 9) ***	–2LL = 690.391, χ^2^ = 299.824 (df = 9) ***	–2LL = 626.565, χ^2^ = 44.495 (df = 9) ***	–2LL = 838.848, χ^2^ = 112.806 (df = 8) ***	–2LL = 239.954, χ^2^ = 68.743 (df = 9)***	–2LL = 226.636, χ^2^ = 22.561 (df = 9) **	–2LL = 383.578, χ^2^ = 72.145 (df = 9) ***	–2LL = 843.348, χ^2^ = 109.013 (df = 9) ***

Note: *N* = 738. Data were weighted. Reference groups: age group (age 55–64), sex (female), marital status (no spouse), region (northern), residence (urban). Constants are omitted. Ordinal variables included: education and household income. Logistic regression analysis was conducted. –2LL = –2 log likelihood. * *p* < 0.05, ** *p* < 0.01, *** *p* < 0.001.
